# Induction of DUSP14 ubiquitination by PRMT5-mediated arginine methylation

**DOI:** 10.1096/fj.201800244RR

**Published:** 2018-06-19

**Authors:** Chia-Yu Yang, Li-Li Chiu, Chih-Chi Chang, Huai-Chia Chuang, Tse-Hua Tan

**Affiliations:** * Immunology Research Center, National Health Research Institutes, Zhunan, Taiwan;; † Department of Medical Education and Research, Taichung Veterans General Hospital, Taichung, Taiwan; and; ‡ Department of Pathology and Immunology, Baylor College of Medicine, Houston, Texas, USA

**Keywords:** TCR signaling, MKP6, TAB1, TAK1, TRAF2

## Abstract

Dual-specificity phosphatase (DUSP)14 (also known as MAP-kinase phosphatase 6) inhibits T-cell receptor (TCR) signaling and T-cell–mediated immune responses by inactivation of the TGF-β activated kinase 1 binding protein (TAB1)–TGF-β activated kinase 1 (TAK1) complex and ERK. DUSP14 phosphatase activity is induced by the E3 ligase TNF receptor associated factor (TRAF)2-mediated Lys63-linked ubiquitination. Here we report an interaction between DUSP14 and protein arginine methyltransferase (PRMT)5 by proximity ligation assay; similarly, DUSP14 directly interacted with TAB1 but not TAK1. DUSP14 is methylated by PRMT5 at arginine 17, 38, and 45 residues. The DUSP14 triple-methylation mutant was impaired in PRMT5-mediated arginine methylation, TRAF2-mediated lysine ubiquitination, and DUSP14 phosphatase activity. Consistently, DUSP14 methylation, TRAF2 binding, and DUSP14 ubiquitination were attenuated by PRMT5 short hairpin RNA knockdown. Furthermore, DUSP14 was inducibly interacted with PRMT5 and was methylated during TCR signaling in T cells. Together, these findings reveal a novel regulatory mechanism of DUSP14 by which PRMT5-mediated arginine methylation may sequentially stimulate TRAF2-mediated DUSP14 ubiquitination and phosphatase activity, leading to inhibition of TCR signaling.—Yang, C.-Y., Chiu, L.-L., Chang, C.-C., Chuang, H.-C., Tan, T.-H. Induction of DUSP14 ubiquitination by PRMT5-mediated arginine methylation.

Protein arginine methyltransferases (PRMTs) are enzymes that catalyze the transfer of methyl groups from S-adenosyl methionine to the guanidine nitrogen of arginine residues (1). Protein arginine methylation is involved in several cellular processes, including RNA processing/transport, chromatin remodeling, signaling transduction, and DNA repair (2). For example, PRMT5 induces symmetric dimethylation on arginine residues of histone 2A and histone 4, resulting in enhanced expression of several oncogenes and cell growth–related genes in hepatocytes (3). PRMT5 also induces monomethylation and symmetric dimethylation of nucleoplasmin at the Arg187 residue, contributing to embryo development (4). Protein arginine methylation is also a key regulatory event in T-cell receptor (TCR) signaling and T-cell activation (5). CD28 costimulation increases PRMT1 expression, leading to arginine methylation of nuclear factor of activated T-cell–interacting protein 45 (NIP45) in T cells; methylation of NIP45 modulates its interaction with nuclear factor of activated T cells, resulting in enhanced IL-4 and IFN-γ gene expression (5). In addition, arginine methylation of thymocyte cAMP-regulated phosphoprotein by PRMT4 may play a role in T-cell development (6).

Dual-specificity phosphatases (DUSPs) remove phosphate groups from phosphoserine/phosphothreonine and/or phosphotyrosine residues from their substrates (7). The DUSP family phosphatases are key molecular regulators that modulate MAPK signaling and play important functions in many biologic processes, including neuronal differentiation (8), cell motility (9, 10), antiobesity responses (11), and immune responses (12–15). DUSP14 (also known as MAP-kinase phosphatase 6) is a negative regulator of TCR signaling by inhibiting JNK and ERK (16). The activation of ERK, but not its upstream kinase MEK1/2, is enhanced in DUSP14 knockout T cells (13), supporting the finding that DUSP14 directly dephosphorylates ERK *in vitro* (16). DUSP14 is coimmunoprecipitated with TGF-β activated kinase 1 (TAK1) (13, 17) and TAK1-binding protein 1 (TAB1) (13). If TAB1 (55 kDa) is not detected in the coimmunoprecipitation assays (17), it is likely that TAB1 is not properly separated from the Ig heavy chain in the immunoblot. In fact, a direct interaction of DUSP14 with TAB1, but not with TAK1, has been demonstrated using purified proteins and Alpha technology/protein-protein interaction assays (PerkinElmer, Waltham, MA, USA) (13) as well as *in situ* proximity ligation assay (PLA) (in this report). DUSP14 is also reported to directly interact with TAK1 in the pull-down assay using immunopurified proteins (18); however, DUSP14- and TAK1-immunopurified complexes may contain coimmunoprecipitated TAB1 proteins. DUSP14 indirectly interacts with TAK1 through TAB1 (13). Thus, DUSP14 inhibits T-cell activation during TCR signaling by dephosphorylating TAB1, leading to inactivation of TAK1, IKK, and JNK (13). We further demonstrated that DUSP14 is Lys63-linked ubiquitination by the E3 ligase TNF receptor associated factor (TRAF)2, and this modification stimulates its phosphatase activity (19). Here we report a novel DUSP14-interacting protein, PRMT5, which methylated DUSP14 at arginine 17, 38, and 45 residues. PRMT5-mediated arginine methylation positively regulated DUSP14-TRAF2 interaction, DUSP14 ubiquitination, and DUSP14 phosphatase activity, leading to inactivation of TAB1, IKK, JNK, and ERK.

## MATERIALS AND METHODS

### Plasmids

Myc-DUSP14, Flag-DUSP14, Flag-DUSP14 (C111S) mutant, Flag-TAB1, Flag-TAK1 (13), Flag-DUSP14 (K103R) mutant (19), and Flag-TRAF2 plasmids (20) were previously described. Human DUSP14 cDNA was used for these DUSP14 constructs. Flag-DUSP14 (3R→K) mutant and Flag-DUSP14 (Q29N, T31S) mutant plasmids were generated by PCR mutagenesis. Myc-TAB1 plasmid was constructed by subcloning TAB1 cDNA into the pCMV6-AC-Myc vector (OriGene Technologies, Rockville, MD, USA). Flag-PRMT5 plasmid was constructed by subcloning PRMT5 cDNA into the pCMV6-AC-Flag vector (OriGene Technologies). The PRMT5 short hairpin RNA (shRNA) #1 and #2 constructs were obtained from the National RNAi Core Facility (Taiwan); PRMT5 shRNA #1 and #2 target sequences are 5′-GCCCAGTTTGAGATGCCTTAT-3′ and 5′-GCGTTTCAAGAGGGAGTTCAT-3′, respectively.

### Antibodies and purified proteins

Anti-DUSP14 antibody was generated by immunization of rabbits with the murine DUSP14 peptide ^181^IPDVYEKESRHLMPYWGI^198^ (corresponding to human DUSP14 protein sequence ^181^VPDVYEKESRHLMPYWGI^198^) (13). Anti–phospho-ERK and TAB1 antibodies were reported previously (13). Anti–phospho-JNK antibody (2155-1) was from Epitomics (Cambridge, MA, USA). Anti-TRAF2 (sc-7346) and anti-ubiquitin (sc-8017) antibodies were from Santa Cruz Biotechnology (Dallas, TX, USA). Anti-HA antibody (05-904) was from Upstate Biotechnology (Lake Placid, NY, USA). Anti-PRMT5 antibody (2252) was from Cell Signaling Technology (Danvers, MA, USA). Anti–methyl-arginine antibody (ab5394) was from Abcam (Cambridge, MA, USA). Anti-Flag (clone M2), anti-Myc (clone 9E10), and anti-Actin (#A5441) antibodies were from MilliporeSigma (Burlington, MA, USA). Anti-CD3 (clone OKT3) antibody was from BioLegend (San Diego, CA, USA). Flag-tagged DUSP14 proteins and Myc-tagged PRMT5 proteins were purified from HEK293T cell lysates using anti-tag antibodies and then eluted using Flag and Myc peptides, respectively.

### Alpha technology protein-protein interaction assay

HEK293T cells were cotransfected with Myc-DUSP14 and Flag-PRMT5 for 24 h. The cells were lysed in lysis buffer (50 mM Tris-HC1, 125 mM NaC1, 5% glycerol, 0.2% NP40, 1.5 mM MgC1_2_, 25 mM NaF, 1 mM Na_3_VO_4_). The cell lysates were incubated with anti-Myc beads for 60 min and then incubated with anti-Flag beads for another 60 min. The Alpha signals from each pair of anti-Flag and anti-Myc beads in proximity (<200 nm) (21) were determined by an EnVision 2104 Multilabel Reader (PerkinElmer).

### *In situ* PLA

PLA assays (22) were performed using the Duolink *In Situ* Red Starter Kit (MilliporeSigma) according to the manufacturer’s instructions. Briefly, Flag-DUSP14 plus Myc-PRMT5, Myc-DUSP14 plus Flag-TAK1, Myc-DUSP14 plus Flag-TAB1, or Flag-TAK1 plus Myc-TAB1 coexpressing HEK293T cells were incubated with anti-Flag (Biorbyt, Cambridge, United Kingdom) and anti-Myc (MilliporeSigma) antibodies, followed by rabbit- and mouse-specific secondary antibodies conjugated with oligonucleotides (PLA probes). After ligation and amplification reactions, the PLA signals from each pair of PLA probes in proximity (<40 nm) were visualized as individual red dots by a fluorescence microscope (DM2500; Leica Microsystems, Buffalo Grove, IL, USA).

### *In vitro* methylation assay

Methylation assays were carried out at 37°C for 1 h in 30 µl of buffer containing 50 mM Tris (pH 7.5), 0.1 mM EDTA, 50 mM NaC1, 0.8 mM S-adenosyl methionine, purified Flag-PRMT5 proteins, and purified Flag-DUSP14 wild-type or mutant proteins. Reactions were stopped by boiling in SDS sample buffer; proteins were resolved by SDS-PAGE and then immunoblotted with specific antibodies.

### Liquid chromatography–mass spectrometry

Sample preparation and liquid chromatography–tandem mass spectrometry (MS) analysis were performed as previously described (19, 23).

### Cell transfection, T-cell stimulation, and *in vitro* phosphatase assay

These DUSP14 experiments were used or performed as previously described (13). Cell lines (HEK293T and Jurkat J-TAg T cells) were used as previously described (20).

### Statistical analysis

All experiments were repeated at least 3 times. Bar graphs are presented as mean ± sem; the mean ± sem values were calculated using SPSS v.19 software (IBM, Armonk, NY, USA).

## RESULTS

### DUSP14 is methylated at Arg17, Arg38, and Arg45 residues by the arginine methyltransferase PRMT5

**Figure 1:**
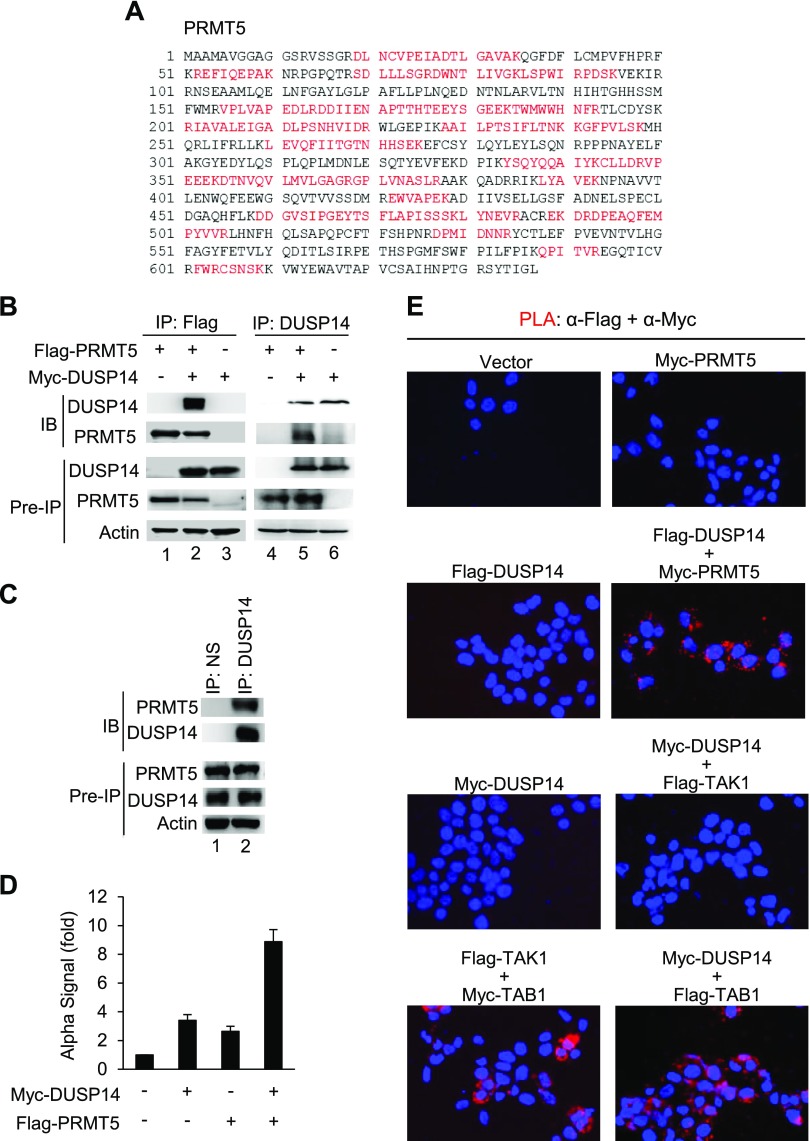
PRMT5 interacts with and methylates DUSP14. *A*) The sequence of PRMT5. Several human PRMT5 peptides (indicated in red) were identified by MS. *B*) DUSP14 interacted with PRMT5. Coimmunoprecipitation (IP) and immunoblotting (IB) analyses of DUSP14 and PRMT5 proteins in lysates of HEK293T cells transfected with empty vector or PRMT5 plasmid with or without DUSP14 plasmid. *C*) Interaction of endogenous DUSP14 with PRMT5 proteins in HEK293T cells was determined by coimmunoprecipitation assays. NS, normal serum. *D*) The interaction between Myc-DUSP14 and Flag-PRMT5 proteins was determined by Alpha technology/protein-protein interaction assay. *E*) PLA showed Flag-DUSP14/Myc-PRMT5, Myc-DUSP14/Flag-TAB1, and Flag-TAK1/Myc-TAB1 interactions, but not Myc-DUSP14/Flag-TAK1 interaction, in HEK293T cells. Each red dot represents an interaction (<40 nm). The cell nucleus was stained with DAPI (blue). Data shown are representative of 3 independent experiments.

To search for novel regulators of DUSP14, we applied a proteomics approach to identify DUSP14-interacting proteins. HEK293T cells were transfected with Flag-DUSP14; DUSP14-associated proteins were coimmunoprecipitated with anti-Flag antibody and then fractionated by SDS-PAGE. Specific protein bands from silver-stained SDS-PAGE gels were excised and digested with trypsin, followed by MS analyses. We found that a protein with an *M*_r_ of about 70 kDa coimmunoprecipitated with DUSP14; this protein was identified as the protein arginine methyltransferase PRMT5 by MS (**Fig. 1*A***). We next confirmed the interaction between DUSP14 and PRMT5 in HEK293T cells by reciprocal coimmunoprecipitation assays. HEK293T cells were cotransfected with Myc-DUSP14 and Flag-PRMT5. The cell lysates were immunoprecipitated with either anti-Flag antibody (Fig. 1*B*, lanes 1–3) or anti-DUSP14 antibody (Fig. 1*B*, lanes 4–6), followed by immunoblotting analysis. The interaction between DUSP14 and PRMT5 was detected by reciprocal immunoprecipitations (Fig. 1*B*). Endogenous DUSP14 and PRMT5 proteins also interacted with each other in HEK293T cells determined by coimmunoprecipitation assays (Fig. 1*C*). Alpha technology is a bead-based assay used to study protein-protein interactions up to 200 nm in solution (21). HEK293T cells were transfected with Myc-DUSP14 and Flag-PRMT5. The cell lysates were incubated with anti-Myc acceptor beads and then anti-Flag donor beads. Alpha interaction signals between Myc-tagged DUSP14 and Flag-tagged PRMT5 proteins were detected (Fig. 1*D*). To further demonstrate the interaction between DUSP14 and PRMT5 proteins *in vivo*, we performed *in situ* PLA, which detects 2 molecules in proximity (<40 nm) in cells with paired antibody-conjugated probes (22). The data showed an interaction between DUSP14 and PRMT5 proteins in HEK293T cells cotransfected with Flag-DUSP14 and Myc-PRMT5 (Fig. 1*E*), suggesting that DUSP14 directly binds to PRMT5. The PLA data also showed a direct interaction of Myc-tagged DUSP14 proteins with Flag-tagged TAB1 proteins but not with Flag-tagged TAK1 proteins.

**Figure 2:**
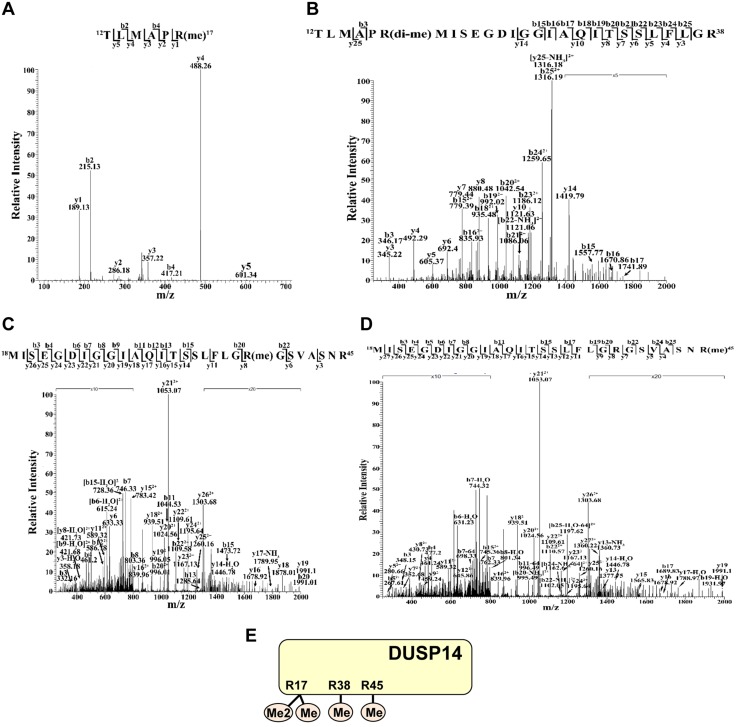
DUSP14 is methylated at arginine 17, 38, and 45 residues. The MS/MS fragmentation spectra of the tryptic peptides of DUSP14 contain the methylation modifications of Arg17 (*A*, *B*), Arg38 (*C*), and Arg45 (*D*) residues. Arg38 (*C*) and Arg45 (*D*) were monomethylated, whereas Arg17 was either monomethylated (*A*) or dimethylated (*B*). *E*) Schematic diagram of methylated residues on the DUSP14 protein. Me, monomethylation; Me2, dimethylation. Data shown are representative of 2 independent experiments.

Given that PRMT5 is an arginine methyltransferase, we studied whether DUSP14 is arginine methylated using MS. DUSP14 proteins were immunoprecipitated from DUSP14-overexpressed HEK293T cells and characterized by MS analysis. Three arginine residues (Arg17, Arg38, and Arg45) of DUSP14 were identified to be methylated (**Fig. 2**). Arg38 and Arg45 were monomethylated, whereas Arg17 was both monomethylated (Fig. 2*A*) and dimethylated (Fig. 2*B*).

**Figure 3:**
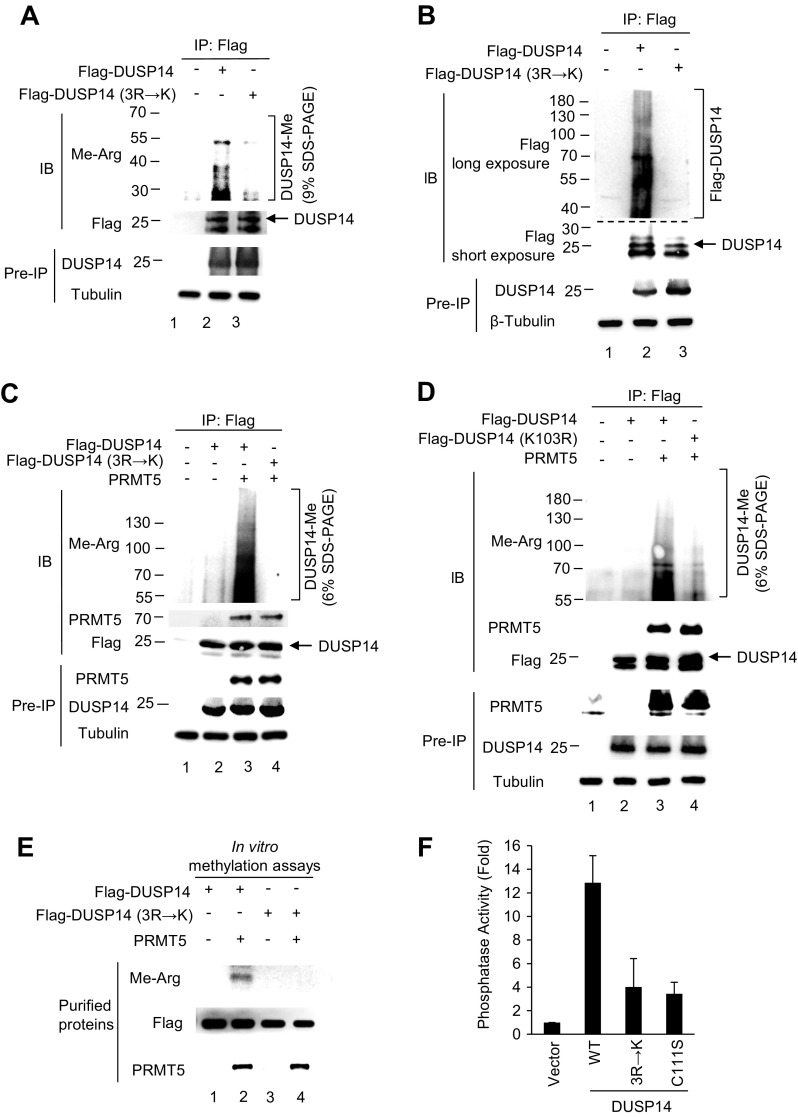
PRMT5-induced DUSP14 methylation is required for its phosphatase activity. *A*) Arginine 17, 38, and 45 residues of DUSP14 were methylated *in vivo.* Flag-tagged DUSP14 wild-type or mutant (3R→K) was transfected into HEK293T cells. Flag-tagged DUSP14 was immunoprecipitated with anti-Flag antibody and then immunoblotted with anti–methyl-arginine antibody. Methylated DUSP14 proteins were fractionated on 9% SDS-PAGE. *B*) Multiple ladder bands detected in *A* were posttranslationally modified DUSP14 proteins. Multiple ladder bands of Flag-tagged DUSP14, but not DUSP14 mutant (3R→K), proteins were detected by immunoprecipitation and then immunoblotting, both using anti-Flag antibody. *C*) Methylation of DUSP14 proteins was enhanced by PRMT5 overexpression. Myc-PRMT5 plus either Flag-DUSP14 wild-type or mutant (3R→K) were cotransfected into HEK293T cells. Flag-tagged DUSP14 was immunoprecipitated with anti-Flag antibody and then immunoblotted with anti–methyl-arginine, anti-PRMT5, or anti-Flag antibody. Methylated DUSP14 proteins were fractionated on 6% SDS-PAGE; the <50 kDa methyl-arginine bands were run off the gel. *D*) The high-MW ladder bands were due to DUSP14 ubiquitination. Myc-PRMT5 plus either Flag-DUSP14 wild-type or mutant (K103R) were cotransfected into HEK293T cells. Flag-tagged DUSP14 proteins were immunoprecipitated with anti-Flag antibody and then immunoblotted with anti–methyl-arginine, anti-PRMT5, or anti-Flag antibody. Methylated DUSP14 proteins were fractionated on 6% SDS-PAGE; the <50 kDa methyl-arginine bands were run off the gel. *E*) DUSP14 was methylated by PRMT5 *in vitro.* Purified Flag-tagged DUSP14 wild-type, Flag-tagged DUSP14 mutant (3R→K), and Myc-tagged PRMT5 proteins were used for *in vitro* methylation assay. PRMT5-methylated DUSP14 wild-type, but not DUSP14 mutant (3R→K), proteins were detected by immunoblotting using anti–methyl-arginine antibody. *F*) DUSP14 methylation was essential for its phosphatase activity. Flag-DUSP14 wild-type, mutant C111S, or 3R→K was transfected into HEK293T cells. DUSP14 proteins were immunoprecipitated with anti-Flag antibody, and DUSP14 phosphatase activity was determined by *in vitro* phosphatase assay. Arrowhead indicates the DUSP14 protein (upper band of the doublet). Data shown are representative of 3 independent experiments.

To confirm that these identified arginine residues are direct targets for methylation by PRMT5, the DUSP14 mutant (3R→K) was generated in which Arg17, Arg38, and Arg45 residues were altered to lysine residues. Flag-tagged DUSP14 wild-type or mutant (3R→K) was transfected into HEK293T cells. The methylation of immunoprecipitated DUSP14 was detected by immunoblotting using an anti–methyl-arginine antibody, which has been successfully used for detecting the arginine methylation of proteins (24). The methylation of wild-type DUSP14 was detected as multiple ladder bands, whereas the methylation of DUSP14 mutant (3R→K) was significantly reduced (**Fig. 3*A***). These multiple ladder bands were also detected by anti-Flag antibody (Fig. 3*B*), indicating that the ladder bands were indeed posttranslationally modified DUSP14 proteins. Next, we studied whether PRMT5 induces DUSP14 methylation. PRMT5 plus either Flag-DUSP14 wild-type or mutant (3R→K) were cotransfected into HEK293T cells. DUSP14 methylation was further enhanced by PRMT5 overexpression (Fig. 3*C*, lane 3), whereas the PRMT5-induced DUSP14 methylation was decreased by DUSP14 3R→K mutation (Fig. 3*C*, lane 4). We cannot rule out the possibility that the 3R→K mutation causes misfolding or other disruption that destroys the enzyme. Nevertheless, the binding of DUSP14 to PRMT5 detected by coimmunoprecipitation was not affected by DUSP14 3R→K mutation (Fig. 3*C*). In addition, multiple ladder bands of methylated DUSP14 proteins detected in Fig. 3A, C using anti–methyl-arginine antibody were reduced by DUSP14 ubiquitination-deficient mutation (K103R) (Fig. 3*D*); this result suggests that the high-MW ladder bands are due to DUSP14 ubiquitination. We further studied whether PRMT5 directly methylates DUSP14 *in vitro* by methylation assays using purified PRMT5 and DUSP14 proteins (Fig. 3*E*). When wild-type DUSP14 proteins were incubated with PRMT5 proteins, we found that DUSP14 was methylated by PRMT5 (Fig. 3*E*, lane 2). Methylation of DUSP14 mutant (3R→K) was significantly reduced compared with that of wild-type DUSP14 (Fig. 3*E*, lanes 2 and 4). These results support that DUSP14 is methylated by PRMT5 *in vitro* and *in vivo*.

DUSP14 is a phosphatase that dephosphorylates phosphoserine/phosphothreonine and/or phosphotyrosine residues of its substrates. To determine the functional relevance of DUSP14 methylation, we examined DUSP14 phosphatase activity by *in vitro* phosphatase assays. HEK293T cells were transfected with Flag-DUSP14 wild-type, C111S mutant, or 3R→K mutant. The cell lysates were immunoprecipitated with anti-Flag antibody. The anti–Flag-DUSP14 immunoprecipitates were subjected to *in vitro* phosphatase assays using p-nitrophenyl phosphate as the substrate. For DUSP14 mutant (C111S), the Cys-to-Ser substitution resulted in a reduction of phosphatase activity (Fig. 3*F*). Similarly, the phosphatase activity of DUSP14 mutant (3R→K) was significantly reduced compared with that of wild-type DUSP14 (Fig. 3*F*). This result indicates that at least 1 of the 3 methylated arginine residues is required for DUSP14 phosphatase activity. In addition, the Arg45 residue is not present in the mouse sequence (the residue in the mouse sequence at this position is Trp45). This could be important for differential regulation of DUSP14 activity in human and mouse.

### DUSP14 methylation regulates its ubiquitination

**Figure 4:**
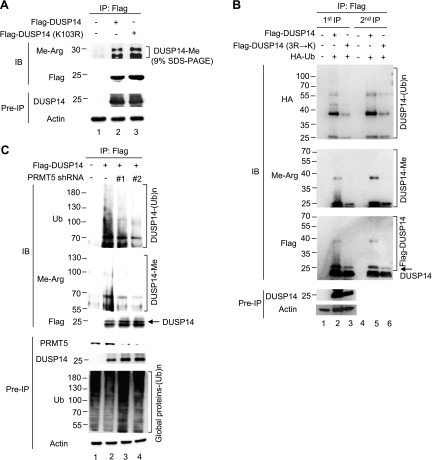
DUSP14 methylation is essential for its ubiquitination. *A*) Methylation of DUSP14 ubiquitination-defective mutant (K103R) was comparable to that of wild-type DUSP14. Flag-DUSP14 wild-type or ubiquitination-defective mutant (K103R) was transfected into HEK293T cells. Flag-tagged DUSP14 proteins were immunoprecipitated from transfected cells with anti-Flag antibody and then immunoblotted with anti–methyl-arginine antibody. Methylated DUSP14 proteins were fractionated on 9% SDS-PAGE. *B*) DUSP14 methylation regulated its ubiquitination. Flag-DUSP14 wild-type or mutant (3R→K) was cotransfected with HA-ubiquitin into HEK293T cells. Flag-tagged DUSP14 was immunoprecipitated with anti-Flag antibody (first IP); half of the first anti-Flag immunoprecipitates were denatured, followed by a second round of immunoprecipitation (second IP) with anti-Flag antibody. Immunoprecipitates were immunoblotted with anti-HA, anti–methyl-arginine, or anti-Flag antibody. The protein markers used in this figure were different from those in *A* and *C*. Methylated DUSP14 proteins were fractionated on 9% SDS-PAGE. *C*) DUSP14 methylation was reduced by PRMT5 shRNA knockdown. Flag-DUSP14 were cotransfected with PRMT5 shRNA #1 or #2 into HEK293T cells. The cell lysates were immunoprecipitated with anti-Flag antibody and then immunoblotted with anti-ubiquitin (Ub), anti–methyl-arginine, or anti-Flag antibody. The cell lysates were also directly immunoblotted with anti-ubiquitin antibody to detect global ubiquitination in cells. Methylated DUSP14 proteins were fractionated on 6% SDS-PAGE; the <50 kDa methyl-arginine bands were run off the gel. Arrowhead indicates the DUSP14 protein (upper band of the doublet). Data shown are representatives of 3 independent experiments.

Because we found that DUSP14 was modified by both methylation and ubiquitination, we studied the potential crosstalk between these 2 posttranslational modifications. First, we tested whether DUSP14 ubiquitination is required for its methylation. Flag-tagged DUSP14 wild-type or ubiquitination-defective mutant (K103R) was immunoprecipitated with anti-Flag antibody from transfected HEK293T cells, followed by immunoblotting with anti–methyl-arginine antibody. We found that DUSP14 wild-type and ubiquitination-defective mutant (K103R) were methylated equally well in cells (**Fig. 4*A***). These results indicate that DUSP14 ubiquitination is not required for DUSP14 methylation by PRMT5. Because DUSP14 may exist as a dimer (25), we cannot rule out the possibility that 1 monomer could be ubiquitinated and the other is arginine methylated.

We next studied whether DUSP14 methylation regulates its ubiquitination. HEK293T cells were transfected with HA-ubiquitin plus either Flag-DUSP14 wild-type or methylation-defective mutant (3R→K). Flag-tagged DUSP14 proteins were immunoprecipitated with anti-Flag antibody from the transfected cells and then immunoblotted with anti-HA antibody for detecting ubiquitinated proteins. Wild-type DUSP14 was ubiquitinated, whereas methylation-defective mutant (3R→K) was only slightly ubiquitinated (Fig. 4*B*, lanes 1–3). To confirm that these ubiquitinated species were indeed DUSP14 proteins, we denatured the anti–Flag-DUSP14 immunoprecipitates to dissociate DUSP14-interacting proteins and then reimmunoprecipitated Flag-DUSP14 using anti-Flag antibody after renaturation. Ubiquitinated bands of DUSP14 wild-type were detected in the second anti–Flag-DUSP14 immunoprecipitates (Fig. 4*B*, lane 5), indicating that DUSP14 itself is ubiquitinated. The reduced ubiquitination of DUSP14 methylation–defective mutant (3R→K) (Fig. 4*B*, lane 6) suggests that DUSP14 methylation regulates its ubiquitination. To further study whether PRMT5 is responsible for methylation and subsequent ubiquitination of DUSP14, we depleted PRMT5 with shRNA and then assessed the levels of DUSP14 methylation and ubiquitination. Knockdown of PRMT5 by each shRNA decreased both methylation and ubiquitination of DUSP14 (Fig. 4*C*). We performed immunoblotting using anti-ubiquitin antibody to show no global disruption of ubiquitination in cells by PRMT5 shRNA knockdown (Fig. 4*C*, bottom panel). These data suggest that PRMT5-mediated DUSP14 methylation regulates DUSP14 ubiquitination.

### DUSP14 methylation induces its binding to TRAF2

**Figure 5:**
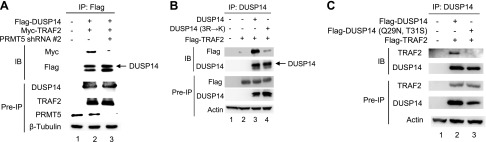
DUSP14 methylation induces its binding to TRAF2. *A*) The interaction between DUSP14 and TRAF2 was inhibited by PRMT5 shRNA knockdown. Myc-TRAF2, Flag-DUSP14, and PRMT5 shRNA #2 were cotransfected into HEK293T cells. The cell lysates were immunoprecipitated with anti-Flag antibody and then immunoblotted with anti-Myc or anti-Flag antibody. *B*) DUSP14 methylation–defective mutant did not bind to TRAF2. Flag-TRAF2 plus either Flag-DUSP14 wild-type or mutant (3R→K) was transfected into HEK293T cells. The cell lysates were immunoprecipitated with anti-DUSP14 antibody and then immunoblotted with anti-Flag or anti-DUSP14 antibody. *C*) DUSP14 methylation enhanced its interaction with the E3 ligase TRAF2. Flag-TRAF2 plus either Flag-DUSP14 wild-type or mutant (Q29N/T31S) were cotransfected into HEK293T cells. The cell lysates were immunoprecipitated with anti-DUSP14 antibody and then immunoblotted with anti-TRAF2 or anti-DUSP14 antibody. Arrowhead indicates the DUSP14 protein (upper band of the doublet). Data shown are representative of 3 independent experiments.

DUSP14 Lys63-linked ubiquitination is induced by the E3 ligase TRAF2 (19). After examination of DUSP14 protein sequence, we found that DUSP14 contains a potential TRAF2-binding motif (PXQXT, where X represents any amino acid) (26), ^27^IAQIT^31^, near the putative DUSP14 methylation sites. Thus, we studied whether PRMT5-mediated DUSP14 methylation regulates TRAF2 binding to DUSP14 using PRMT5 shRNA and coimmunoprecipitation assays. HEK293T cells were cotransfected with Myc-TRAF2, Flag-DUSP14, and either PRMT5 shRNA #2 or empty vector. The data showed that the interaction between DUSP14 and TRAF2 was reduced by PRMT5 shRNA knockdown (**Fig. 5*A***). Moreover, DUSP14 methylation–defective mutant (3R→K) showed drastically reduced interaction with TRAF2 in transfected HEK293T cells (Fig. 5*B*, lane 4). These results suggest that DUSP14 methylation promotes its binding to TRAF2. To confirm that the aforementioned ^27^IAQIT^31^ motif within DUSP14 indeed mediates TRAF2 binding, we generated a putative TRAF2-binding–deficient DUSP14 mutant by mutating Gln29 and Thr31 to Asn and Ser, respectively. Compared with DUSP14 wild-type, DUSP14 mutant (Q29N, T31S) did not interact with TRAF2 (Fig. 5*C*, lane 3). Collectively, these results indicate that the PRMT5-mediated methylation of DUSP14 promotes the TRAF2-mediated ubiquitination of DUSP14.

### DUSP14 methylation and subsequent ubiquitination are enhanced by TCR signaling in T cells

**Figure 6:**
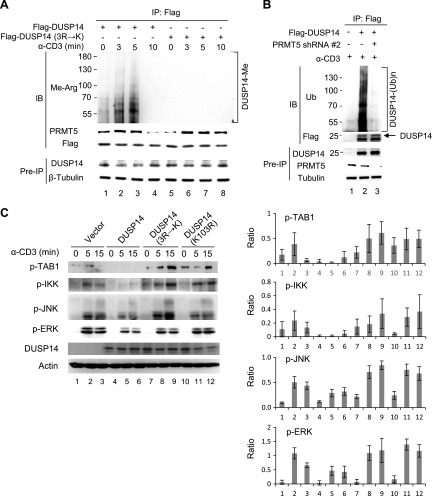
Methylation and ubiquitination of DUSP14 in T cells are enhanced by TCR signaling. *A*) DUPS14 was inducibly interacted with PRMT5 upon anti-CD3 stimulation. Flag-DUSP14 was transfected into Jurkat (J-TAg clone) T cells and then stimulated with anti-CD3 antibody. Cell lysates were immunoprecipitated with anti-Flag antibody, followed by immunoblotting with anti–methyl-arginine, anti-PRMT5, or anti-Flag antibody. PRMT5 immunoblotting was performed by reprobing using the methyl-arginine immunoblot membrane with anti-PRMT5 antibody. *B*) PRMT5-induced DUSP14 methylation regulates its ubiquitination during TCR signaling. Flag-DUSP14 plus either PRMT5 shRNA or vector were transfected into Jurkat (J-TAg clone) T cells. The transfected cells were then stimulated with anti-CD3 antibody for 15 min. DUSP14 proteins were immunoprecipitated with anti-Flag antibody and then immunoblotted with anti-ubiquitin (Ub) or anti-Flag antibody. *C*) Flag-DUSP14 wild-type or mutant (3R→K) was transfected into Jurkat (J-TAg clone) T cells. The transfected cells were then stimulated with anti-CD3 antibody for 15 min. Phosphorylation of TAB1, IKK, JNK, and ERK was detected by immunoblotting using individual anti–phospho-specific antibodies (left panel). The relative phosphorylation levels of 3 experiments were determined by densitometry analysis (right panel). Means ± sem of 3 independent experiments are shown. Data shown are representative of 3 independent experiments.

**Figure 7:**
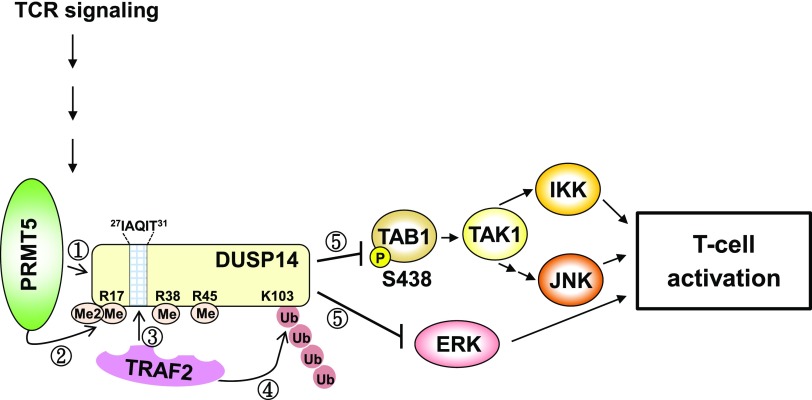
Model for DUSP14 activation by methylation-induced ubiquitination. Upon TCR stimulation, PRMT5 methylates arginine 17, 38, and 45 residues of DUSP14. DUSP14 contains a putative TRAF2-binding motif, ^27^IAQIT^31^, which is close to 3 putative methylation sites. Arginine-methylated DUSP14 then recruits the E3 ligase TRAF2, which in turn induces Lys63-linked ubiquitination at Lys103 residue of DUSP14. Methylation and subsequent ubiquitination stimulate the phosphatase activity of DUSP14. DUSP14 indirectly interacts with TAK1 through TAB1 (13). Activated DUSP14 dephosphorylates TAB1, leading to sequential inactivation of TAK1 and IKK/JNK. Activated DUSP14 also directly dephosphorylates and inhibits ERK. Collectively, DUSP14 methylation and subsequent ubiquitination activate DUSP14 phosphatase activity, leading to attenuation of T-cell activation.

We further studied whether TCR signaling induces the PRMT5-mediated DUSP14 methylation in T cells using coimmunoprecipitation assays. Jurkat (J-TAg clone) T cells were transfected with either Flag-DUSP14 wild-type or mutant (3R→K). Stimulation with anti-CD3 antibody induced the interaction between DUSP14 and PRMT5 in transfected Jurkat T cells (**Fig. 6*A***, second panel); the increase of DUSP14-PRMT5 interaction was concomitant with the induction of DUSP14 methylation (Fig. 6*A*, top panel). In contrast, TCR-induced DUSP14 methylation was abolished by the 3R→K mutation, whereas the inducible interaction between DUSP14 and PRMT5 was unaffected by the 3R→K mutation (Fig. 6*A*, lanes 5–8). These results suggest that PRMT5 may mediate DUSP14 methylation during TCR signaling. Next, we studied whether PRMT5-mediated DUSP14 methylation also regulates its ubiquitination in activated T cells using PRMT5 shRNA knockdown. Jurkat (J-TAg clone) cells were cotransfected with Flag-DUSP14 and either PRMT5 shRNA #2 or empty vector, followed by anti-CD3 stimulation. The ubiquitination levels of immunoprecipitated DUSP14 proteins were examined by immunoblotting analysis using anti-ubiquitin antibody. Knockdown of PRMT5 by shRNA abolished ubiquitination of DUSP14 in transfected Jurkat T cells upon anti-CD3 stimulation (Fig. 6*B*, lane 3). These results suggest that methylation of DUSP14 by PRMT5 is required for DUSP14 ubiquitination during TCR signaling. We also investigated whether mutations of the methylation and ubiquitination sites of DUSP14 affect its phosphatase activity during TCR signaling. Jurkat (J-TAg clone) T cells were transfected with Flag-DUSP14 wild-type, methylation-defective mutant (3R→K), and ubiquitination-defective mutant (K103R). The activation of TAB1, IKK, JNK, or ERK was determined by immunoblotting analysis using individual anti–phospho-specific antibodies. DUSP14 wild-type reduced the activation of TAB1, IKK, JNK, and ERK in transfected Jurkat T cells upon anti-CD3 stimulation (Fig. 6*C*, lanes 4–6), whereas DUSP14 methylation–defective mutant (3R→K) and ubiquitination-defective mutant (K103R) did not (Fig. 6*C*, lanes 7–12). Taken together, these results support that PRMT5-mediated methylation and subsequent TRAF2-mediated ubiquitination of DUSP14 promote the activation of its phosphatase activity in T cells during TCR signaling (**Fig. 7**).

## DISCUSSION

Here we report that DUSP14 was methylated at arginine 17, 38, and 45 residues by the arginine methyltransferase PRMT5. Furthermore, PRMT5-mediated arginine methylation of DUSP14 promotes its ubiquitination, which may lead to DUSP14 activation during TCR signaling. The regulation of DUSP14 ubiquitination by its methylation may be due to enhancement of the interaction between the methylated DUSP14 and the E3 ligase TRAF2. Notably, DUSP14 contains the putative TRAF2-binding motif ^27^I-A-Q-I-T^31^, which is close to 3 putative methylation sites (arginine 17, 38, and 45 residues). Our data also showed that double mutations (Q29N, T31S) on the putative TRAF2-binding motif of DUSP14 abolished its TRAF2 binding. Given the above observations, we propose a model (Fig. 7) that PRMT5 inducibly interacts with and methylates DUSP14 at arginine 17, 38, and 45 residues, which in turn facilitate TRAF2 binding to the neighboring residues Gln29/Thr31, leading to an induction of DUSP14 ubiquitination and phosphatase activity. Activated DUSP14 then dephosphorylates TAB1, leading to sequential inactivation of TAK1 and IKK/JNK. Activated DUSP14 also directly dephosphorylates and inhibits ERK, but not MEK1/2 (13, 16). Collectively, DUSP14 methylation and subsequent ubiquitination are induced during TCR signaling, resulting in T-cell inactivation.

Arginine methylation can modulate protein-protein interactions by disrupting hydrogen bonding or by increasing hydrophobicity (1). PRMT1-mediated NIP45 methylation facilitates its interaction with nuclear factor of activated T cells (5). Methylation of receptor interacting protein 140 reduces its interaction with histone deacetylase 3 and facilitates its interaction with exportin 1 (24). Thus, besides enhancing TRAF2 binding by methylation, it is possible that PRMT5-induced DUSP14 methylation may enhance its interaction with other DUSP14-interacting proteins during cell signaling.

Protein methylation also regulates protein stability through inducing Lys48-linked ubiquitination (27–29). For example, the lysine methyltransferase Set9-induced Smad7 methylation stimulates its Lys48-linked ubiquitination, leading to proteasomal degradation of Smad7 (27). In contrast, PRMT6 methylates the HIV-1 viral protein Tat and inhibits Tat ubiquitination, resulting in enhancement of Tat protein stability through Lys48-linked ubiquitination (29). Similarly, krüppel-like factor 4 arginine methylation by PRMT5 inhibits krüppel-like factor 4 ubiquitination and prevents subsequent protein turnover (28). Instead of regulating protein stability, we found that arginine methylation sequentially regulates Lys63-linked ubiquitination and phosphatase activity of DUSP14. Besides the protein methylation–regulated ubiquitination, protein methylation induces acetylation. The methylation of p53 at the Lys372 residue induces acetylation at adjacent lysine residues, resulting in activation of p53 transcriptional activity (30). In addition, protein methylation regulates phosphorylation (31, 32). Arginine methylation of the DNA/RNA-binding protein heterogeneous nuclear ribonucleoprotein K inhibits its phosphorylation by PKCδ, resulting in enhancement of heterogeneous nuclear ribonucleoprotein K–mediated cell apoptosis (31). Furthermore, PRMT1-mediated arginine methylation of Axin induces its phosphorylation by glycogen synthase kinase 3 β, leading to decreased ubiquitination and subsequent stabilization of Axin (32). It would be interesting to study whether methylation or ubiquitination of DUSP14 regulates other posttranslational modifications, such as phosphorylation and acetylation of DUSP14. For example, arginine methylation of DUSP14 by PRMT5 may regulate phosphorylation of DUSP14, which in turn stimulates TRAF2-mediated ubiquitination of DUSP14.

The activity of PRMT5 is regulated by phosphorylation (3). The serine-threonine kinase rho-associated kinase phosphorylates PRMT5 at the Thr80 residue, which induces PRMT5 activity (3). In contrast, myosin phosphatase dephosphorylates Thr80 phosphorylation of PRMT5 and abolishes PRMT5 activity (3). Our data showed that the PRMT5-DUSP14 interaction, as well as methylation, ubiquitination, and phosphatase activity of DUSP14, were inducible during TCR signaling in T cells. Another DUSP family member, DUSP22, is an important negative regulator that dephosphorylates Lck in the TCR signaling turn-off stage, leading to suppression of T-cell responses (14). It would be interesting to study whether methylated DUSP14 may negatively feed-back regulate (dephosphorylate) PRMT5 and may attenuate PRMT5 activity in the turn-off stage of TCR signaling.

## Abbreviations

DUSP: dual-specificity phosphatase
MS: mass spectrometry
NIP45: nuclear factor of activated T-cell–interacting protein 45
PLA: proximity ligation assay
PRMT: protein arginine methyltransferase
shRNA: short hairpin RNA
TAB: TGF-β activated kinase binding protein
TAK: TGF-β activated kinase
TCR: T-cell receptor
TRAF: TNF receptor associated factor
